# Development and Validation of an In‐House Indirect ELISA for Serological Detection of *Gallibacterium anatis* in Laying Hens Using Local Field Isolates

**DOI:** 10.1155/vmi/9467618

**Published:** 2026-09-16

**Authors:** Raudatul Jannah, Agustin Indrawati, Ryan Septa Kurnia, Christian Marco Hadi Nugroho, Muhammad Ade Putra, Sri Murtini

**Affiliations:** ^1^ Master Student of Animal Biomedical Sciences, School of Veterinary Medicine and Biomedical Sciences, IPB University, Bogor 16680, Indonesia, ipb.ac.id; ^2^ Division of Medical Microbiology, School of Veterinary Medicine and Biomedical Sciences, IPB University, Bogor 16680, Indonesia, ipb.ac.id; ^3^ Animal Health Diagnostic Unit, PT Medika Satwa Laboratoris, Bogor 16166, Indonesia

**Keywords:** antibody, *Gallibacterium anatis*, indirect ELISA, laying hens, poultry, serodiagnosis

## Abstract

*Gallibacterium anatis* is an emerging opportunistic pathogen associated with respiratory and reproductive tract infections in poultry, causing reduced egg production and economic losses in laying hens. Serological detection is important for surveillance; however, no validated in‐house enzyme‐linked immunosorbent assay (ELISA) based on local Indonesian isolates is available. This study aimed to develop and validate an indirect ELISA for detecting antibodies against *G. anatis* in laying hens. Five archived isolates were characterized by PCR targeting gyrB, gtxA, and flfA virulence‐associated genes, followed by growth rate and biofilm formation analyses. Isolate B116, which showed the highest growth rate and strongest biofilm‐forming ability, was selected for antigen preparation. Four antigen extraction methods, including heat treatment at 56°C and 96°C, capsular extraction, and sonication, were evaluated to determine the best coating antigen. Checkerboard titration was performed to optimize antigen concentration, serum dilution, and HRP‐conjugated rabbit anti‐chicken IgG dilution. Cross‐reactivity was assessed using positive sera against *Pasteurella multocida*, *Avibacterium paragallinarum, Escherichia coli*, *Salmonella sp.*, and *Clostridium perfringens*. The optimal ELISA conditions were 7.11 μg/mL coating antigen concentration, 1:200 serum dilution, and 1:8000 conjugate dilution. The cutoff values were 0.156 and 0.189 using mean ± 2SD and mean ± 3SD. Compared with PCR, the in‐house ELISA showed 85.0% sensitivity and 94.7% specificity. Field serum testing revealed the highest seropositivity in laying hens aged 29–48 weeks. The developed in‐house ELISA demonstrated good diagnostic performance and may serve as a practical tool for large‐scale serological surveillance and epidemiological monitoring of *G. anatis* infection in poultry farms.

## 1. Introduction

Poultry meat and eggs are considered significant and sustainable animal protein sources worldwide, and their consumption by humans has increased significantly, particularly in developing countries [1, 2]. Currently, about 1500 billion eggs and 120 million tons of poultry meat are produced annually worldwide [3]. Despite enormous growth in the poultry sector, it continues to be hampered by numerous factors, with disease being a major one, which caused high morbidity and mortality in acute form [4, 5]. One of the bacteria commonly linked with infection in poultry production systems is *Gallibacterium anatis* [6]. The bacterium has been reported in several European, African, and Asian countries [7]. *G. anatis* belongs to the family Pasteurellaceae, an important opportunistic pathogen and an emerging disease in poultry [8]. In a previous study [9], *G. anatis* was associated with pathological conditions, including salpingitis, peritonitis, septicemia, enteritis, and respiratory tract lesions.

*G. anatis* is a nonmotile, Gram‐negative rod‐shaped or pleomorphic bacterium distributed solitary or in pairs. Colonies of the bacteria are grayish and nontransparent with a strong hemolytic zone on blood agar, but eventually translucent at the periphery and have a butyrous consistency. They are circular, raised with an entire margin smooth, glossy, and non–spore forming [10]. *G. anatis* is capable of growing in microaerophilic environments, exhibiting positive catalase, oxidase, and phosphatase results, but negative for indole and urea [11]. Furthermore, *G. anatis* is separated into two biovars: *G. anatis haemolytica* and *G. anatis anatis,* whereas *G. anatis* biovar *haemolytica* is regarded as having a significant pathogenic potential [12]. *G. anatis* typically transmits infection through horizontal dissemination via the respiratory route and vertical transmission via transovarian infection [13]. Isolation from different chicken organs of *G. anatis* demonstrated the bacterium’s systemic circulation and spread from its natural habitat [14]. According to the study conducted by [15], the isolation of *G. anatis* from calves suggests a potential zoonotic relevance of this bacterium. In addition, the first reported case of *G. anatis* bacteremia in an immunocompromised woman further highlights its possible zoonotic potential.

Early diagnosis, prevention, and control are crucial for managing disease outbreaks and preventing economic losses [16]. The gold standard for detecting *G. anatis* is the isolation of the pathogen; however, this method is time‐consuming and prone to overgrowth from secondary bacterial contamination. Substantial antigenic diversity of *G. anatis* makes it challenging to prevent its adverse effects using traditional test [13]. Serological techniques could be helpful in early monitoring of infectious diseases by detecting specific antibodies for a particular pathogen, which guide the necessary prophylactic measures to control the outbreak [1]. The enzyme‐linked immunosorbent assay (ELISA) is a potent immunological assay for detecting specific antibodies or antigens. It is based on an immunoassay that integrates the specific interaction between antigen and antibodies and the catalysis between enzyme and substrate [17]. To the best of our knowledge, no prior reports have been published about the *G. anatis* serological assay from local Indonesian isolates. The present study aimed to develop an antigen‐based indirect ELISA as a rapid, economical diagnostic instrument and to evaluate its comparative performance for the serodetection of *G. anatis* antibodies.

## 2. Materials and Methods

### 2.1. Ethical Approval

The research protocols used in this study were approved by the IPB University Animal Ethics Committee under approval number 298/KEH/SKE/II/2025 on 12 February 2025.

### 2.2. Bacterial Isolates Characterization

The *G. anatis* isolates utilized in this investigation were obtained from clinically affected chickens showing respiratory symptoms. The isolates were collected in 2023 from respiratory, reproductive organ, and trachea swabs. Five samples consisted of archived isolates obtained from breeder and layer chicken farms in Sukabumi Regency, West Java, Indonesia (approximately 6.92°S, 106.93°E) and Bogor Regency, West Java, Indonesia (approximately 6.60°S, 106.80°E). The isolate was identified based on colony morphology, Gram staining, and standard biochemical tests, followed by molecular confirmation using polymerase chain reaction (PCR) that targeted the *G. anatis–*specific gene [18]. The phenotypic characterization encompassed the evaluation of growth rate and biofilm‐forming ability, both of which are recognized as indicators of bacterial persistence and virulence.

### 2.3. Growth Rate and Biofilm Formation Analysis

Bacteria were previously cultured in sheep blood agar at 37°C overnight. The tubes containing media were placed in BioSan LV Personal Bioreactor RTS‐1C (Riga, Latvia) to maintain the temperature of the tubes containing media at a precise 0.1°C. The RTS‐1C was used to collect OD850 nm measurements at 30‐min intervals. To propagate the culture, one loop of bacteria colonies from the blood agar culture was transferred to a new tube containing 20 mL of fresh media [19]. Biofilm formation was observed by culturing in a 96‐well microtiter plate for 12, 24, 36, 48, 60, and 72 h at 37°C. The concentration that yielded the greatest reduction in biomass was used for subsequent assays. Following treatment, the wells were delicately rinsed, stained with crystal violet, and the bound dye was quantified. Phosphate‐buffered saline (PBS)–treated wells served as negative controls. All experiments were performed in five replicative samples. The microplate reader was employed to measure the remaining percentage of biofilm biomass through absorption at a 595‐nm wavelength [20, 21].

### 2.4. Pathogenicity Assessment

The isolate selected for pathogenicity assessment in this study was the isolate exhibiting the highest virulence profile, the fastest growth rate, and the strongest biofilm‐forming capacity. The study employed 30 specific antibody‐negative (SAN) ISA Brown chickens, which were randomly divided into three groups. The chickens in Group 1 were intranasally challenged with 0.2 × 10^9^ CFU/mL of *G. anatis* suspended with PBS. Group 2, which was uninfected and housed in the same cage, served as the transmission group, while Group 3 was not challenged and served as the negative control. Chickens were confined in isolated conditions under negative pressure and on dense litter, with ad libitum access to feed and water.

Clinical symptoms were scored according to the method described by Paudel et al. [22] and observed on Days 6, 8, 10, 12, and 14 postinfection. A score of 0 indicated normal chickens without clinical signs; 1, mild nasal discharge or swelling of sinus infraorbitalis; 2, nasal discharge along with swelling of sinus infraorbitalis; and 3, severe swelling of sinus infraorbitalis with or without conjunctivitis. Tracheal swabs were collected on Days 6, 10, 14, and 18 postinfection and directly plated onto blood agar. The blood was collected aseptically from the wing vein on Day 18 postinfection from all treatment groups. The blood was then centrifuged to obtain serum for in‐house ELISA testing [22].

### 2.5. Antigen Preparation

Four different *Gallibacterium anatis* antigens were evaluated as ELISA coating antigens, namely, heat‐treated antigens (56°C and 96°C), capsular antigen, and sonicated antigen. Briefly, *G. anatis* archive isolates were grown in brain heart infusion broth (BHI) at 37°C for 6 h. Subsequently, 0.1 mL of the bacterial suspension was spread onto a blood agar plate and incubated at 37°C for 18 h. Bacterial colonies were harvested, resuspended in 5 mL of PBS, and placed in a dry bath at 56°C and 96°C for 1 h, respectively. For capsular treatment, bacterial colonies were suspended in 2.5% sodium chloride and agitated in a water bath at 37°C for 4.5 h at 80 rpm. The antigen was sonicated for 30 min at 40.000 Hz after bacterial colonies were resuspended in normal saline. All antigens were centrifuged at 4000 rpm for 1.5 h after undergoing multiple treatments [23]. The supernatant was collected, and the protein concentration was determined by UV–vis spectrophotometry (Genesys, Thermo Scientific, USA) at 260 and 280 nm. Bovine serum albumin was used as a standard. Stock antigen preparations were stored at −20°C for further utilization.

### 2.6. Serum Sample Origin

Serum samples were obtained from 4‐week‐old brown layer chickens. Separately, the concentration of the *G. anatis* bacterial suspension used for antigen preparation was determined through UV spectrophotometry, and inactivation was performed by sonication for 30 min. The injection was administered intramuscularly with 1 mL of bacterial suspension in BHI. The second injection (booster) was prepared using a rotor–stator homogenizer (Ultra‐Turrax T 50 digital, IKA Werke, Germany), incorporating 30% total antigen and 70% oil‐based adjuvant (ISA 71 VG MontanideTM, Seppic, France) at the manufacturer’s 30:70 ratio recommendation. The control group received 1 mL of sterile BHI via the same route. Blood samples were obtained from three groups: the control group, the prevaccinated groups, and the second vaccination (booster) group, 4 weeks after the initial vaccination [24]. Blood samples were centrifuged at 3000 × g for 10 min at 4°C and preserved at −20°C for further analysis.

The antibody responses and the in‐house ELISA’s capacity to distinguish between positive and negative serum samples were evaluated using a known‐positive serum. The optical density (OD) values were subsequently transformed into sample‐to‐positive (S/P) ratios, and the ELISA threshold value (cutoff point) was determined as the mean OD of known negative sera plus 2 or 3 standard deviations (SDs) [25].

### 2.7. ELISA Procedure

The plates were coated with 100 μL of antigen in coating buffer (0.05 M carbonate‐bicarbonate buffer [pH 9.6]) and incubated overnight at 4°C. After three times washing with PBS‐T (pH 7.3), the plate was blocked with 150 μL of TBS‐C (pH 7.3) and incubated for 1 h at room temperature. Following three rounds of incubation and rinsing, 100 μL of each serum dilution was added in accordance with the plate design and incubated at room temperature for 1 h. Afterward, 100 μL of rabbit anti‐chicken IgG‐horseradish peroxidase (HRP) conjugate was added and incubated at room temperature for 1 h, followed by three times washing. Substrate solution containing 100 μL 2,2′‐azino‐bis (3‐ethylbenzothiazoline‐6‐sulfonic acid)) (ABTS) dissolved in citrate buffer (pH 5.0) and H_2_O_2_ was added, and this was then incubated in the dark at room temperature for 20 min [26]. The OD at 405 nm was measured using an AMR‐100 Microplate Reader (Hangzhou Allsheng).

### 2.8. Optimization of Coating Antigen Candidate

The indirect ELISA conditions were optimized by checkerboard titration using serial dilutions of the extract antigen, the antibody primer, and the conjugate. Briefly, several antigen extracts of *G. anatis* were coated onto the microplate at dilutions of 1:50, 1:100, and 1:200. The control serum was diluted to 1:100, and the HRP‐conjugated rabbit anti‐chicken IgG antibody was diluted to 1:4000 and 1:8000. In order to achieve the most effective antigen‐extracting treatment, the OD value derived from the method was calculated and presented as ± ratio. Antigen coating was also assessed based on protein concentration from several treatments. Dilutions that gave the maximum value between positive and negative sera (P/N) were selected for the optimal antigen coating concentration and sera dilution [27].

### 2.9. Determination of Cross‐Reactivity Optimization of Serum Dilution

In order to identify the most effective antigen types for the detection of antibodies against *G. anatis*, the specificity of the ELISA was assessed using sera that were positive for *G. anatis* and those that were positive for *Pasteurella multocida*, *Avibacterium paragallinarum*, *Escherichia coli*, *Salmonella sp*., and *Clostridium perfringens* but negative for *G. anatis*.

### 2.10. Optimization of Serum and Secondary Antibody Dilution

For the sensitivity analysis, the ELISA was performed using a two‐fold serial dilution of six serum samples at 1:100, 1:200, 1:400, 1:800, 1:1600, 1:3200, 1:6400, and 1:12,800 (diluted in TBS‐Tween casein).

Secondary antibodies were diluted in PBS‐T (1:2000, 1:4000, 1:8000, and 1:16.000) and subsequently added to each well. The plates were incubated for 1 h at room temperature. Afterward, ABTS was added to each well, and the plate was incubated [28].

### 2.11. Determination of the Sensitivity and Specificity of the In‐House ELISA

The diagnostic sensitivity and specificity of the in‐house ELISA were determined according to the methods described by Hurisa et al. and Samad et al. [29, 30], using conventional PCR as the reference method. PCR amplification was performed in a 25‐μL reaction mixture containing 12.5 μL of MyTaq HS Red Mix (Meridian Bioscience, USA), 1 μL of the forward primer 1133fgal (5′‐TATTCTTTGTTACCARCGG‐3′), 1 μL of the reverse primer 114r (5′‐GGTTTCCCCATTCGG‐3′), 8.5 μL of nuclease‐free water, and 2 μL of DNA template. *Gallibacterium anatis* was confirmed by amplification of a species‐specific 1030‐bp fragment using the primer pair 1133fgal and 114r. The PCR results were used as the reference standard for evaluating the diagnostic performance of the in‐house ELISA. Diagnostic sensitivity and specificity were calculated by comparing the ELISA results with the PCR results, as presented in Table 1.

**TABLE 1:** Determination of the sensitivity and specificity of the in‐house ELISA.

Table 2 × 2		ELISA		Total
		Positive (+)	Negative (−)	
PCR	Positive (+)	TP	FN	
	Negative (−)	FP	TN	

*Note:* TP = number of positive samples in both in‐house and PCR; FN = number of samples that are positive to in‐house but negative in PCR; FP = number of samples that are negative in in‐house ELISA but positive in PCR; TN = number of negative samples in both in‐house ELISA and PCR.

### 2.12. Determination of the Cutoff Value

Under the optimized ELISA conditions, negative *G. anatis* serum samples were examined. The average S/P value and SD were calculated. S/P = (OD405 of sample to be tested – average OD405 of negative samples)/(average OD405 of positive samples – average OD405 of negative samples). The cutoff value of the ELISA was calculated from the mean S/P of sera negative samples plus 2 or 3 SDs.

### 2.13. Serologic Test in a Field Serum Sample by In‐House ELISA

Fifty‐six serum samples were obtained from commercial chicken farms located in Sukabumi, Indonesia. The samples originated from multiple housing units level based on the chicken’s age. Each housing represented a single age group, including 10 weeks from breeder, 8, 14, 19, 29, 36, and 48 weeks from layer. Blood samples from each chicken were aseptically collected into 3‐mL tubes for serum separation, stored at 4°C, and then centrifuged within 12 h of collection at 2500 rpm for 10 min to separate the serum. The collected sera were kept at −20°C until further use [16]. The reaction conditions used during the in‐house ELISA optimization procedures were identical to those used to test all serum samples using in‐house ELISA.

### 2.14. Data Analysis

Data were analyzed using GraphPad Prism 9 (GraphPad Software, San Diego, CA, USA). Descriptive statistics were used to summarize the indirect ELISA OD values, which are presented as mean ± SD. The indirect cutoff value was established as the mean OD of the negative control sera plus two (mean + 2SD) and three (mean + 3SD). Diagnostics performance, including sensitivity, specificity, positive predictive value (PPV), negative predictive value (NPV), and positive likelihood ratio (LR+), was calculated using PCR as the reference standard. The corresponding 95% confidence intervals (95% CIs) were also determined for each diagnostic performance parameter.

## 3. Results

### 3.1. Bacterial Isolates Characterization

**FIGURE 1:**
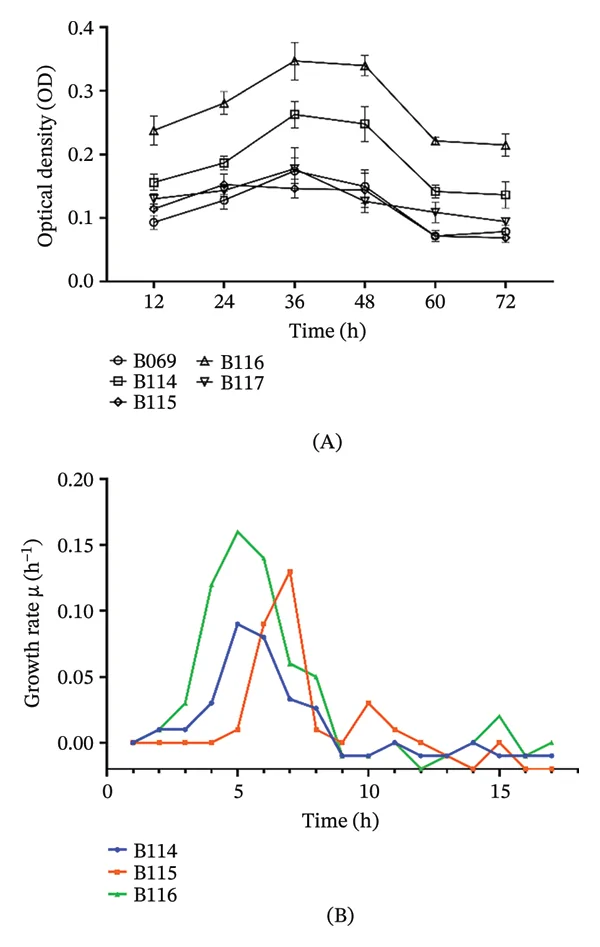
Biofilm formation and growth rate of *G. anatis* isolates. (A) Biofilm formation of five *G. anatis* isolates (B069, B114, B115, B116, and B117) evaluated by the crystal violet assay. Biofilm biomass was quantified by measuring the optical density (OD) at the indicated incubation times (12, 24, 36, 48, 60, 72 h); (B) growth rate (μ, h^−1^) of selected isolates over time, showing variations in bacterial growth dynamics during the incubation period. The *x*‐axis represents incubation time (h), whereas the *y*‐axis indicates the specific growth rate (μ, h^−1^).

Analysis of five *G. anatis* isolates revealed differential patterns of *gyrB*, *gtxA*, and *flfA* gene presence (Table 2). Only two isolates contained all three genes, whereas the remaining three isolates harbored only two genes, with *gyrB* detected in all isolates. Isolates carrying the *flfA* gene were further evaluated for their growth characteristics, as *flfA* is a well‐established factor in bacterial adhesion. Biofilm formation analysis revealed that isolate B116 exhibited the highest biofilm‐forming capacity, as indicated by OD measurements (Figure 1). Consistently, this isolate demonstrated a higher growth rate compared to the other isolates assessed in this study.

**TABLE 2:** Distribution of selected virulence genes (*gyrB, gtxA,* and *flfA*) among *G. anatis* isolates obtained in this study.

Isolate code	Virulence genes		
	*gyrB*	*gtxA*	*flfA*
MSL B069	✓	✓	—
MSL B114	✓	—	✓
MSL B115	✓	✓	✓
MSL B116	✓	✓	✓
MSL B117	✓	✓	—

### 3.2. Assessing Pathogenicity

**FIGURE 2:**
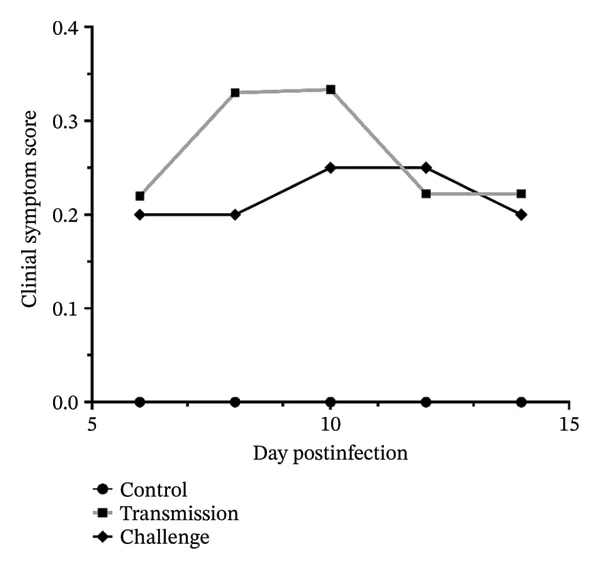
Clinical symptom scores of ISA Brown chickens following experimental *Gallibacterium anatis* infection. Clinical signs were evaluated on Days 6, 8, 10, 12, and 14 postinfection using the clinical scoring system described by Paudel et al. [22]. Chickens were assigned to three experimental groups: challenge (intranasally inoculated with *G. anatis*), transmission (noninoculated chickens housed with the challenged birds), and control (noninoculated, nonexposed chickens). Data are presented as the mean clinical symptom score of each group (*n* = 10 chickens per group).

The number of poultry exhibiting clinical signs and their scores following the challenge is given in Figure 2. Clinical signs first appeared at 2 days postinfection (dpi) with *G. anatis*. Increased clinical symptoms were observed on Day 6 postinfection and increased from Day 10 to Day 14. In the transmission animal group, clinical symptoms increased on Days 8 and 10 and decreased on Days 12 and 14. No fatalities occurred in any of the treated groups during the observation period, as evidenced by daily clinical symptom observations. No animals with severe symptoms were identified, so based on these results, the survival rate during the observation period was 100% and the clinical condition generally showed an improving trend. From the pathogenicity challenge assessment, S/P values were obtained from the challenge group, ranging from 0.047 to 0.204. The cutoff values at mean ± 2SD and mean ± 3SD were 0.156 and 0.189, respectively.

### 3.3. Optimize Antigen Coating

The ± ratio from several treatments between positive and negative serum samples is displayed in Table 3. The optimal treatment for antigen extraction was sonication at an antigen dilution of 1:200. The resulting ratio in the sonicated antigen was 9.49, with a lower concentration of 0.84 μg/mL. The positive serum absorbance of the sonicated antigen was 1.705, which is 9.06 times greater than the negative absorbance value. In the sonicated antigen, the resulting ratio at a lower conjugate concentration (1:8000) gave a value that was still equivalent to the higher concentration, while the resulting ratios from other treatments were significantly smaller.

**TABLE 3:** The checkerboard titration.

Ratio (±)	Antigen concentration (μg/mL)											
		96			56			Capsular			Sonication	
Conjugate	**3.38**	**1.69**	**0.84**	**3.38**	**1.69**	**0.84**	**3.38**	**1.69**	**0.84**	**3.38**	**1.69**	**0.84**
1:4000	10.59	10.83	10.83	8.28	9.21	9.58	0.80	9.14	9.39	9.88	10.09	9.94
1:8000	9.31	8.68	8.84	3.85	8.87	8.14	8.34	8.35	8.44	8.92	9.49	*9.07*

*Note:* The bold values represent the different antigen concentrations evaluated in the checkerboard titration. Specifically, 3.38, 1.69, 0.84 μg/mL correspond to antigen dilutions of 1:50, 1:100, and 1:200, respectively. The italic value (9.07) indicates the condition selected as the optimal working condition based on the checkerboard titration, using an antigen concentration of 0.84 μg/mL (1:200) and a conjugate dilution of 1:8000.

### 3.4. Cross‐Reactivity Assessment

**FIGURE 3:**
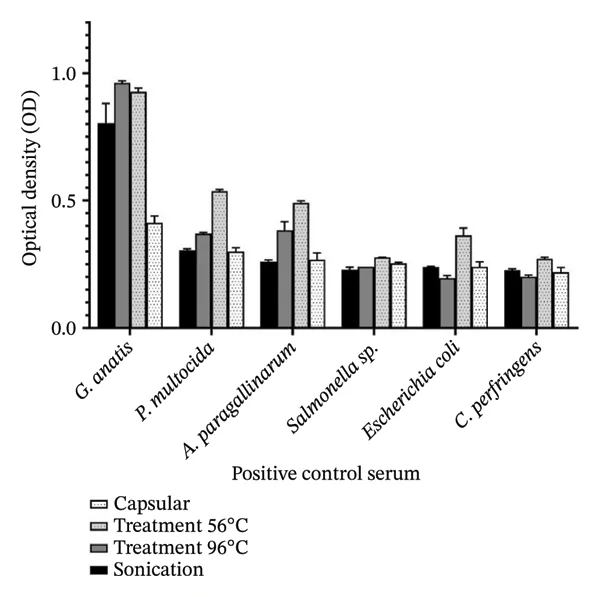
Indirect ELISA reactivity of positive control serum against from different bacterial species prepared under various treatment conditions. Serum reactivity was assessed under capsular, heat treatment (56°C and 96°C), and sonication conditions. The *y*‐axis represents the ELISA optical density (OD) measured at 450 nm, with higher OD values indicating stronger antibody reactivity. Bars represent mean OD values with error bars indicating standard deviation.

Positive serum samples against other pathogens were employed to identify the cross‐reactivity of the *G. anatis* ELISA, as illustrated in Figure 3. According to the difference calculations between treatments for each bacterium, a relatively high distance was observed for the antigen treated at 96°C, with values of 0.59 and 0.58 for *P. multocida* and *A. paragallinarum*, respectively. This distance was even higher for the subsequent test bacteria. In the sonicated antigen, the differences obtained were 0.50 and 0.54 for *P. multocida* and *A. paragallinarum*, respectively, and were within the same range for the subsequent test bacteria.

The difference values obtained were not higher than those for the 96°C heat‐treated antigen; however, compared with previous test results, including concentration, the sonicated antigen demonstrated better quality as a coating antigen and was utilized in the subsequent stage.

### 3.5. Optimization of Primary Antibody and Secondary Antibody

**FIGURE 4:**
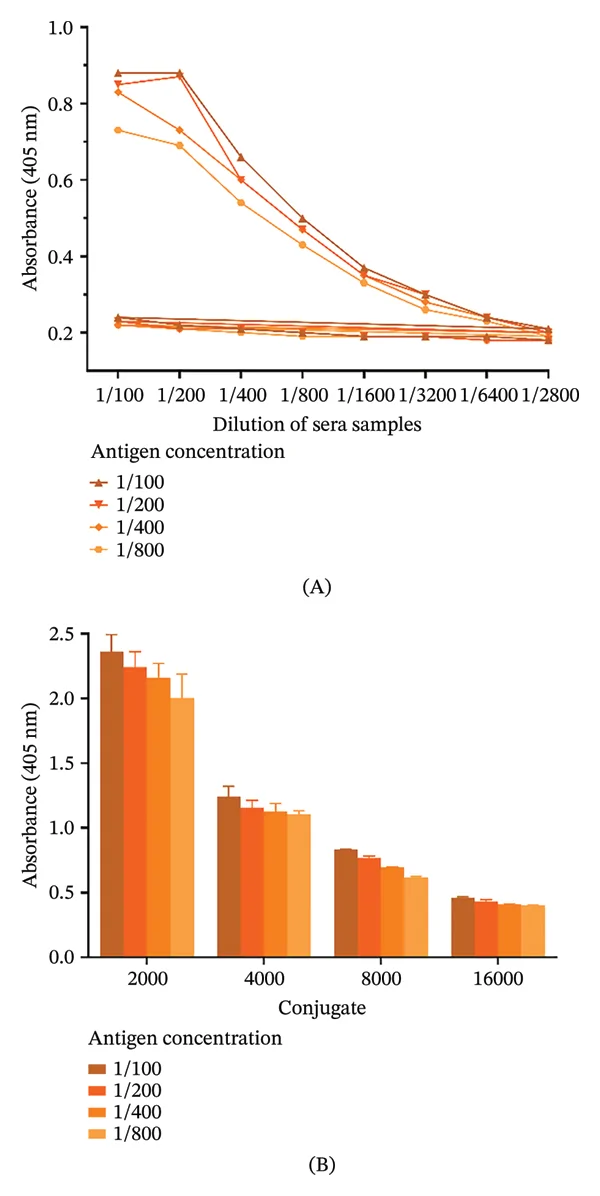
Optimization of antigen concentration, serum dilution, and conjugate dilution for the indirect ELISA using checkerboard titration. (A) Indirect ELISA showing absorbance (405 nm) at varying serum dilutions and antigen concentrations (1:100–1:800). (B) Effect of conjugate dilution (1:2000–1:16,000) on absorbance values at varying antigen concentrations.

In the in‐house ELISA serum dilution optimization, the positive serum consistently yielded higher values than the negative serum across all antigen concentrations and dilution levels (Figure 4A). In the positive serum, the OD value decreased as the serum dilution increased from 1:100 to 1:12.800. Sensitivity analysis based on the limit of detection (LOD) generated a value of 0.248, corresponding to the standard dilution of the serum sample at 1:3200. Subsequently, the optimal conditions for *G. anatis* ELISA were determined to generate the greatest disparity between the positive and negative sera (P/N). The maximum P/N (4.08) value was obtained at a 1:200 dilution of the antigen and the serum sample (Table 4).

**TABLE 4:** Results of the P/N values at different conditions.

Antigen concentration (μg/mL)		Dilution of primary antibody							
		1:100	1:200	1:400	1:800	1:1600	1:3200	1:6400	1:12,800
14.21	P	0.88	0.88	0.66	0.50	0.37	0.30	0.24	0.21
	N	0.24	0.22	0.21	0.20	0.19	0.19	0.19	0.18
	P/N	3.71	4.07	3.19	2.51	1.92	1.57	1.30	1.15

7.10	P	0.85	0.87	0.60	0.47	0.35	0.30	0.24	0.20
	N	0.23	0.21	0.21	0.20	0.19	0.19	0.18	0.18
	P/N	3.72	4.08	2.93	2.35	1.85	1.60	1.28	1.09

3.55	P	0.83	0.73	0.60	0.47	0.35	0.28	0.24	0.20
	N	0.22	0.21	0.21	0.20	0.19	0.19	0.18	0.18
	P/N	3.69	3.40	2.91	2.39	1.88	1.52	1.28	1.13

1.77	P	0.73	0.69	0.54	0.43	0.33	0.26	0.23	0.19
	N	0.22	0.21	0.20	0.19	0.19	0.19	0.18	0.18
	P/N	3.37	3.24	2.66	2.19	1.76	1.40	1.26	1.08

The secondary antibody optimization was performed to determine the optimal dilution of the HRP‐conjugated rabbit anti‐chicken IgY for the in‐house ELISA. Based on the ELISA and P/N (4.07) value, the optimal dilution was 1:8000, which distinguished the positive and negative sera samples and reduced background signal (Figure 4B).

### 3.6. Cutoff Value

**FIGURE 5:**
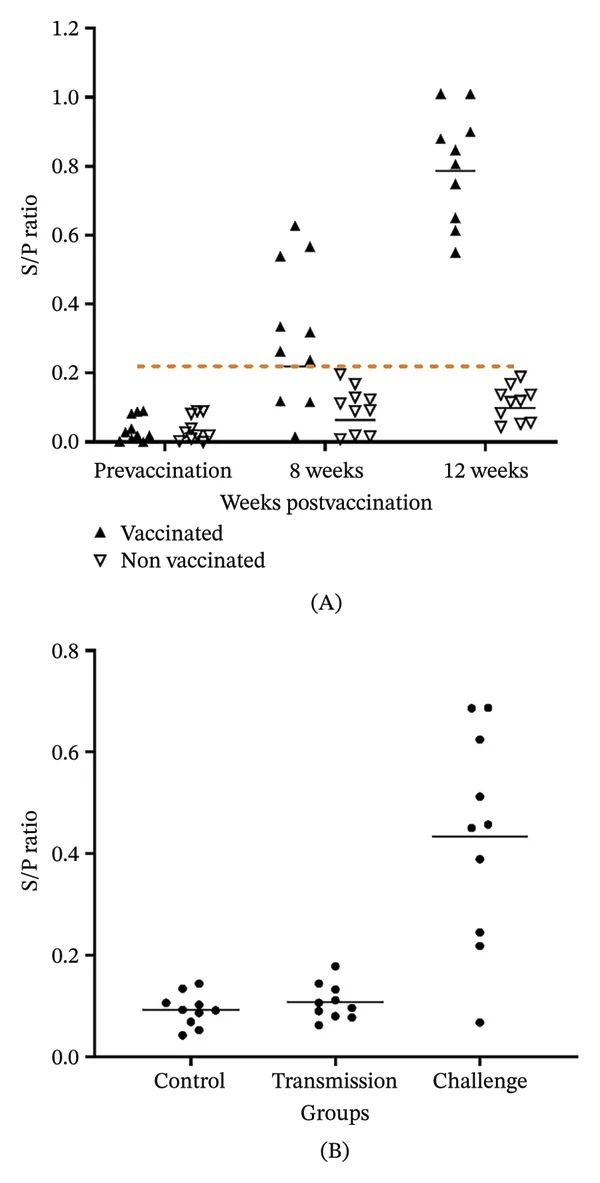
Antibody response measured by S/P ratio in chickens following vaccination and challenge models. (A) S/P ratios of vaccinated and nonvaccinated groups at pre‐vaccination, 8 weeks, and 12 weeks postvaccination. The dashed line indicates the cutoff value. (B) Comparison of S/P ratios among control, transmission, and challenge groups. The mean values are indicated by horizontal lines, while each point denotes an individual sample.

The serum samples from vaccinated animals were used to evaluate the in‐house ELISA, which revealed significant differences between the groups. The S/P ratio for the 4‐week‐old poultry serum was used as a control, and the prevaccination serum had an average S/P ratio of 0.038. Antibody titers increased in several individuals, with the average post‐first vaccination of 0.314. From post‐booster observations at 12 weeks of age, all individuals exhibited substantially higher antibody titers, with an average of 0.801 (Figure 5A). Therefore, the S/P value of 10 negative serum samples ranged from a minimum of 0.008 to a maximum of 0.197. Thus, subjects with an S/P ratio ≤0.222 were classified as seronegative, whereas those with as S/P ratio > 0.222 were classified as seropositive.

### 3.7. Sensitivity and Specificity of In‐House ELISA

Performance analysis of the in‐house ELISA was compared with PCR as the gold standard. The pathogenicity test resulted in 17/39 true positives (TP), 3/39 false negatives (FN), 1/39 false positives (FP), and 18/39 true negatives (TN) (Table 5). These values yielded a sensitivity of 85% (95% CI: 62%–96%) and a specificity of 94.7% (95% CI: 73%–99%). Based on the calculation of PPV and NPV, the results obtained were consistent, which were 94% (95% CI: 72%–99%) and 85% (95% CI: 63%–96%), respectively. The performance of the in‐house ELISA was also assessed using the LR. The LR value obtained was 16.15 (Table 6).

**TABLE 5:** 2 × 2 diagnostic contingency.

Table 2 × 2		In‐house ELISA		Total
		Positive	Negative	
PCR	Positive	17	3	20
	Negative	1	18	19

**TABLE 6:** Validation of in‐house ELISA diagnostic.

	Value (%)	CI 95%
Sensitivity	85	62%–96%
Specificity	94	73%–99%
Positive predictive value	94	72%–99%
Negative predictive value	85	63%–96%
Likelihood ratio (LR+)	16.15	

### 3.8. Field Serum Sample

**FIGURE 6:**
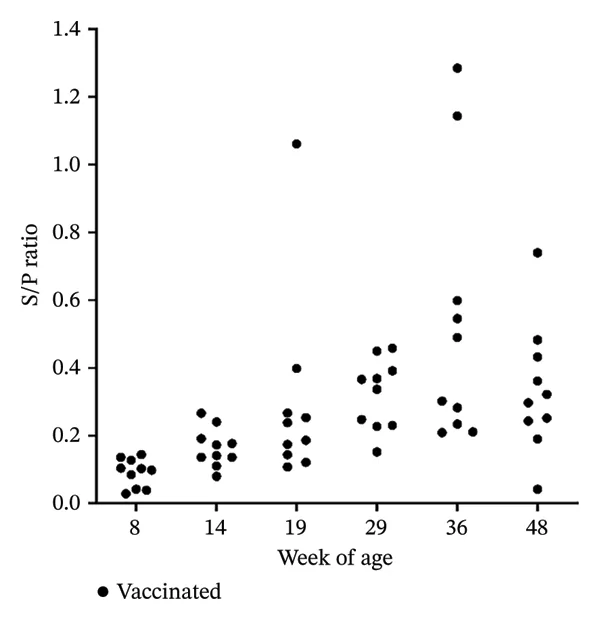
Antibody response (S/P ratio) in field samples from chickens suspected of respiratory disease at various ages (8–48 weeks). Each point represents an individual sample, showing variation in antibody levels among different age categories.

In ELISA testing with field samples, 56 samples of chicken blood serum that indicated respiratory disease were obtained. Serum samples were collected from six different breeding cages for layer chickens. The age range of chickens in the field sample testing was 8, 14, 19, 29, 36, and 48 weeks. The infection status was determined by PCR; 11 samples were confirmed positive for *G. anatis*, while the remaining 45 samples were negative. Chicken serum at 8 and 14 weeks of age had a low average S/P ratio of 0.088 and 0.179, respectively. From the serum of 19‐week‐old chickens, 1 in 10 chickens was found to have high antibody levels against *G. anatis* with an S/P ratio of 1.06. The average S/P ratio of the group was 0.29. Higher variations in S/P ratio values between individuals in the same group were also found in chickens aged 36 and 48 weeks. In the 36‐week‐old cohort, the S/P ratio values were 1.14 and 1.28, with an average of 0.53. Meanwhile, in the 48‐week age group, the S/P ratio for one individual was 0.74, with an average of 0.33 (Figure 6).

## 4. Discussion

The molecular characterization of archived *G. anatis* isolates in this study revealed heterogeneity in the distribution of virulence‐associated genes. All isolates examined in the present study carried the *gyrB* gene, which encodes the B subunit of DNA gyrase, an essential enzyme involved in DNA replication and supercoiling. The existence of *gyrB* in all isolates is consistent with its function as a housekeeping gene that is highly conserved across bacterial species. Due to its genetic stability and essential cellular function, *gyrB* is frequently employed as a molecular marker for bacterial identification and phylogenetic analysis [18].

In contrast, the distribution of the virulence‐associated genes *gtxA* and *flfA* varied among the isolates. The *gtxA* gene encodes an RTX‐like toxin, a major virulence factor of *G. anatis*. This toxin contributes to host cell damage through cytotoxic and hemolytic activity, facilitating bacterial colonization and persistence within host tissues [3]. The *flfA* gene encodes a fimbrial protein involved in bacterial adhesion, a critical early step in host colonization and biofilm formation. Adhesion factors enable the attachment of microbes to epithelial surfaces, thereby establishing stable colonization niches within the host. Previous studies have shown that fimbrial structures play a crucial role in the pathogenicity of *G. anatis*, particularly in enabling its persistence within the reproductive tract of poultry [31].

In accordance with the established biological function of *flfA*, isolates carrying this gene were further evaluated for their growth characteristics and biofilm‐forming capacity. The biofilm formation assay revealed that isolate B116 exhibited the highest OD, indicating the strongest biofilm‐forming ability among the tested isolates. Biofilm formation is a critical survival strategy for numerous bacterial pathogens, as it provides structural protection against environmental stress, host immune responses, and antimicrobial agents [32]. Interestingly, this investigation exhibited a significantly higher growth rate compared to the other isolates assessed. A higher growth rate may provide a competitive advantage for bacterial colonization and dissemination within host tissues. When combined with enhanced biofilm formation, this characteristic has the potential to increase the isolate’s overall virulence.

Therefore, isolate B116 was selected for further analyses that were designed to prepare antigens and evaluate them for serological detection through indirect ELISA. The selection of this isolate was based on its molecular and phenotypic characteristics, including the presence of virulence‐associated genes and its robust biofilm‐forming ability. These characteristics may contribute to the expression of antigenic proteins that are pertinent for antibody detection.

Sonicated antigens showed positive serum absorbance values of 1.705 and 9.06, which were 9.06 and 9.06 times greater than the negative absorbance value. In sonicated antigens, the ratio generated at a lower conjugate concentration (1:8000) gave a value that was still equivalent to a higher concentration, whereas the resulting ratio value was significantly smaller in other treatments. The use of sonicated antigens in the laboratory offers increased safety by reducing the risk of infection, as evidenced by a study conducted by Niloofa et al. [26]. Their findings demonstrated that sonicated antigens can provide a safer alternative to the use of viable *G. anatis serovars* in laboratory assays. In the sonicated antigen, *P. multocida* and *A. paragallinarum* exhibited a difference of 0.50 and 0.54, respectively, in the same range of values against the subsequent test bacteria. The value obtained was not significantly different from that of the 96°C heating antigen. However, as evidenced by the concentration results from the prior test, the sonicated antigen provided better coating quality and was employed in the subsequent stage.

Antigen extraction involving heating at 56°C and 96°C in this case releases proteins that are conserved across Gram‐negative species, resulting in significant differences in values compared to those of other pathogenic bacteria. The presence of these conserved proteins may explain the cross‐reactivity observed in in‐house ELISA testing against nonhomologous bacteria. The term “cross‐reactivity” of antibodies refers to the binding of antibodies to epitopes on other antigens, which is caused by low avidity or the presence of identical epitopes on different antigens. Cross‐reactivity was typically observed within antigen groups where the chemical structure or amino acid sequence is evolutionarily conserved. Small differences in amino acid sequences result in diverse and distinct properties that mediate variations in antigen binding, specificity, and biological activity. According to Pors et al. [33], the outer membrane proteins of *G. anatis* and other bacteria from the Pasteurellaceae family contain homologous epitopes that have the potential to induce cross‐reactivity in crude antigen‐based assays. The assay displays a significantly lower specificity against other Gram‐negative poultry pathogens, such as *E. coli* and *Salmonella* spp., and the probability of cross‐reactivity is exceedingly low. Nevertheless, both pathogenic bacteria share similarities, particularly in the cell wall constituent lipopolysaccharide (LPS), exhibiting similar epitopes [34].

The OD values of the positive serum test graph diminish as the serum dilution increases from 1:100 to 1:12,800 (Figure 2). This demonstrates the correlation between antibody titration and lower serum concentrations, which results in a reduced number of specific antibodies available to bind the antigen [35]. At dilutions of 1:100 to 1:800, the signal‐to‐noise ratio differs most significantly between positive and negative sera, suggesting that the test’s sensitivity is optimal. At higher dilutions (≥ 1:1600), the signal from positive sera begins to approach that from negative sera, decreasing sensitivity and the ability to detect specific antibodies. Antigen concentrations of 1:100 and 1:200 yield the highest and most stable OD values in positive sera, potentially serving as optimal concentrations for subsequent testing. Conversely, a lower OD signal is observed at an antigen concentration of 1:800, indicating that the antigen may no longer bind optimally to the antibody.

Positive PCR results indicate the presence of *G. anatis* in all individuals in the infected group. However, not all individuals are able to mount a uniform or effective immune response. Although antigen exposure occurs, the resulting antibody response may vary between individuals and does not necessarily provide protective immunity. PCR detects the presence of the microorganism’s genetic material locally and systemically, but a positive PCR result does not always reflect sufficient antigen exposure to trigger a strong immune response; instead, it may induce clinical symptoms. Localized infection or low inoculum volume due to mechanical reactions of the host upon receiving the bacterial preparation can also lead to an insufficient antigen to induce measurable antibody formation in serum. Generally, PCR and ELISA do not detect the same biological parameters; PCR is more sensitive for detecting pathogen DNA and reflecting active infection. In this study, ELISA was employed to detect antibodies produced after a challenge test, indicating prior infection. Although PCR was used as the gold standard, it remains relevant in this study because the sample status was previously determined by PCR and then revalidated serologically using ELISA [27]. Therefore, differences in results between the two testing methods, such as the presence of FP and FN, do not necessarily indicate deficiencies in the serological test method developed, but rather reflect variations in the phase of infection detected by each test method.

Variations in these field samples indicate exposure to *G. anatis* circulating in the flock. The serum results from field samples suggest that there are variations in the host’s immune response or disparities in natural exposure to *G. anatis* between individual birds. Relatively high antibody levels indicate that some individual birds have quite severe infections. Similar findings were reported by Kristensen et al. [36], who showed that antibody titers against *G. anatis* can vary significantly among individual birds within a population. Chickens at 8 and 14 weeks of age exhibited reduced S/P values and increased consistency among individual birds. This may be related to the tissue tropism of *G. anatis*, which predominantly targets the reproductive tract of laying hens. At this young age, chickens have not yet entered the laying phase, resulting in reduced exposure to reproductive tract infections, limited bacterial colonization, and, consequently, lesser and more uniform antibody responses. In contrast, infection during the laying period has been linked to reproductive disorders and decreased egg production, which may elicit stronger and more unpredictable antibody responses [36].

Seroepidemiological studies not only allow for the evaluation of the effectiveness of previous animal health interventions but also offer insight into the potential risk of future transmission. Serological surveillance has strategic value, particularly for vaccine‐preventable diseases. The results can be utilized to assess the effectiveness of immunization programs and identify individuals who remain susceptible to certain infections and are at risk of triggering outbreaks, enabling more targeted and effective preventive interventions.

## 5. Conclusion

This study demonstrated the molecular heterogeneity of archived *G. anatis* isolates. Among the isolates evaluated, isolate B116 exhibited superior phenotypic traits. This included a greater capacity for growth and enhanced biofilm formation, indicating that the isolate has higher potential for host interaction and persistence. Based on these characteristics, isolate B116 was selected for antigen preparation and further evaluation in serological testing. Among the antigen extraction methods tested, sonication produced the most suitable antigen preparation for indirect ELISA, providing a clear differentiation between positive and negative sera and maintaining adequate sensitivity at lower conjugate concentrations. The in‐house ELISA developed was capable of detecting antibody responses following experimental infection and thereby demonstrating its suitability for the analysis of field serum samples.

The serological analysis of field samples revealed variability in antibody responses among chickens of different ages, suggesting natural exposure to *G. anatis* within the flock. This also underscores the disparities in the immune responses of the host. These findings indicate that the developed ELISA method, which is bolstered by the selected antigen preparation, has potential utility for serological surveillance and epidemiological monitoring of *G. anatis* infections in poultry populations.

## Funding

This research received no specific grant from any funding agency in the public, commercial, or not‐for‐profit sectors.

## Disclosure

All authors have read and approved the final version of the manuscript. The corresponding author had full access to all of the data in this study and takes complete responsibility for the integrity of the data and the accuracy of the data analysis.

The corresponding author affirms that this manuscript is an honest, accurate, and transparent account of the study being reported; that no important aspects of the study have been omitted; and that any discrepancies from the study as planned have been explained.

## Conflicts of Interest

The authors declare no conflicts of interest.

## Data Availability

The data that support the findings of this study are available from the corresponding author upon reasonable request.
